# Exploring spectroscopic X-ray nano-imaging with Zernike phase contrast enhancement

**DOI:** 10.1038/s41598-022-06827-y

**Published:** 2022-02-21

**Authors:** Yeseul Kim, Jun Lim

**Affiliations:** Pohang Accelerator Laboratory, Pohang University of Science and Technology, Jigokro 127, Pohang, Kyungbuk 37637 Republic of Korea

**Keywords:** X-rays, Characterization and analytical techniques

## Abstract

Spectroscopic full-field transmission X-ray microscopy (TXM-XANES), which offers electrochemical imaging with a spatial resolution of tens of nanometers, is an extensively used unique technique in battery research. However, absorption-based bright-field imaging has poor detection sensitivity for nanoscale applications. Here, to improve the sensitivity, we explored spectroscopic X-ray nano imaging with Zernike phase contrast (ZPC-XANES). A pinhole-type Zernike phase plate, which was optimized for high-contrast images with minimal artifacts, was used in this study. When the absorption is weak, the Zernike phase contrast improves the signal-to-noise ratio and the contrast of images at all energies, which induces the enhancement of the absorption edge step. We estimated that the absorption of the samples should be higher than 2.2% for reliable spectroscopic nano-imaging based on XANES spectroscopy analysis of a custom-made copper wedge sample. We also determined that there is a slight absorption peak shift and sharpening in a small absorption sample due to the inflection point of the refractive index at the absorption edge. Nevertheless, in the case of sub-micron sized cathode materials, we believe that better contrast and higher resolution spectroscopic images can be obtained using ZPC-XANES.

## Introduction

X-ray absorption near-edge structure (XANES) spectroscopy is the measurement of transitions from the core electronic states of the metal to the excited electronic states, which provides chemical information, including the oxidation state and local atomic coordination environments^1^. Traditional XANES spectroscopy measures the intensity of X-rays before and after the sample with gas ionization chambers, which are mainly developed for bulk analysis. Although, micro-XANES is available with a synchrotron source^2–5^, the microscale resolution is insufficient for complex systems that require nanoscale chemical state changes or probing local information. Recently, spectroscopic full-field transmission X-ray microscopy (TXM-XANES) has emerged as a new technique for chemical imaging at the nanoscale with two-dimensional (2D) information^6–9^. Owing to the energy tunability, full-field transmission X-ray microscopy (TXM) can generate XANES spectra at each pixel from a stack of images at each energy across the element absorption edge^10^. The energy range of hard X-rays (5–12 keV) covers the K-edge of transition metals used in battery electrodes, including Ni, Co, and Mn; thus, TXM-XANES is considered an essential technique for studying battery electrodes^7–14^. Additionally, it has a high resolution down to $\sim$ 20 nm over a field of view (FOV) of several tens of micrometers, which is suitable for single-particle analysis.

However, as TXM-XANES is a bright-field microscopy based on the absorption contrast, it has poor detection sensitivity for weakly concentrated materials or thin samples where the absorption is weak compared to the background fluctuations. There are several reports on enhancing the contrast by adjusting background fluctuations at absorption TXM, such as adaptive time-dependent intensity normalization^15^, averaged background for normalization^16^, and dynamic intensity normalization using Eigen flat fields^17^. Zernike phase contrast (ZPC) is a well-known contrast enhancement method for TXM to enhance the signal from weak absorption materials^18–20^. The principle of ZPC is that phase changes induced by the sample are transformed into changes in intensity by shifting the phase of the zeroth order by a phase plate (ideally transparent object) with respect to the higher-order spatial frequencies from the sample^21^. Ring-type ZPC requires accurate shape and alignment as it can directly affect image quality^22^. In this context, realigning the phase ring to the corresponding back focal plane according to each energy is a critical limitation for adopting ZPC to conventional on-axis TXM-XANES. Report has also indicated that the detection sensitivity can be improved by combining scanning nanoprobes and fluorescence-yield XANES^23^.

In this study, we propose a simple approach for enhancing the sensitivity of TXM-XANES by implementing the Zernike phase-contrast method. The key point of this method is the use of off-axis illumination and a pinhole-type phase plate, where the zeroth-order frequency focuses on a point and passes through the hole, whereas the phase of the higher-order frequencies shift by $\pi$/2 at the phase plate^24,25^. Because it focuses on a point and the hole at the phase plate has a diameter of a few μm, we can fix the position of the phase plate at the back focal plane of the absorption edge energy (Supplementary Fig. S1). By scanning the energies across the absorption edge of the sample, we can conduct TXM-XANES with the Zernike phase contrast method (ZPC-XANES), which enhances the sensitivity of the weak absorption sample. We present an in-depth study of ZPC-XANES, including a comparison with TXM-XANES. Finally, we discuss future perspectives of this technique.

## Results and discussion

The ZPC-XANES experiment was conducted using the spectroscopic X-ray Nano Imaging Beamline (BL7C) of the Pohang Light Source II (PLS-II, South Korea). This beamline was operated in two modes: zone plate-based full-field transmission X-ray microscopy with Zernike phase^26,27^ and TXM-XANES^28^. Unlike conventional on-axis TXM, BL7C utilizes off-axis illumination, which has the advantages of minimal phase contrast artifacts and easy alignment with the phase plate^25^. Compared to ring-type ZPC, efficiency of pinhole-type ZPC is low since the numerical aperture is small and the thin metal phase plate absorbs high frequency image. Inevitably, there is a disadvantage that the exposure time is rather long.

**Figure 1:**
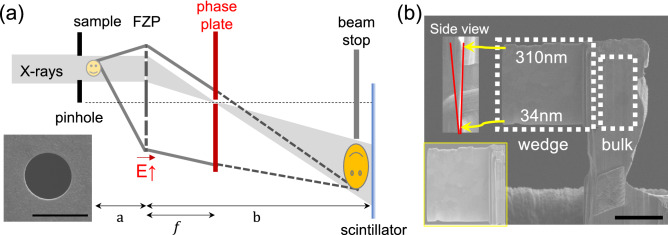
Acquisition of ZPC-XANES. (**a**) Schematic of the off-axis full-field phase contrast XANES spectroscopy setup. The inset shows the 4 μm hole on the Au phase plate. (**b**) Scanning electron microscope (SEM) images of the Cu reference sample with wedge-shaped structures and bulk Cu. The side view shows the thickness where the beam passes through at the wedge structure with the top with a thickness of 310 nm and the bottom with a thickness of 34 nm. The inset shows the adjusted contrast of wedge Cu. The scale bars are 5 μm.

We implemented a gold-phase plate at the back focal plane of the Fresnel zone plate to the existing TXM-XANES setup, as shown in Fig. 1a. The phase of the diffracted beam from the sample (gray solid line) was shifted by $\pi$/2 (gray dashed line) with respect to the direct or un-diffracted (gray shaded region) beam. According to Zernike’s method of phase contrast^21^, these phase changes were transformed into changes in intensity. Because the optimal energy of the phase plate is 9.332 keV, Cu with a K-edge at 8.979 keV, which is close to this energy value, was selected as the sample. To measure the sensitivity of the method, we designed a wedge-shaped Cu sample with a gradual decrease in thickness from the top (310 nm) to the bottom (34 nm) and bulk with 5 μm thickness as shown in Fig. 1b.

**Figure 2:**
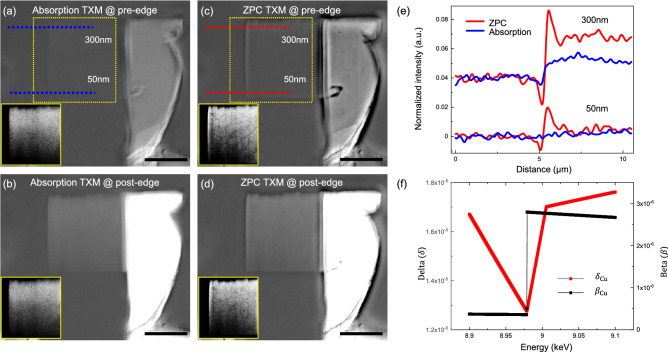
Contrast comparison between absorption and ZPC TXM. (**a**,**b**) Absorption images of the Cu sample from the selected images at pre-edge (8.960 keV) and post-edge (9.060 keV), respectively. (**c**,**d**) phase contrast images of the Cu sample from the selected images at pre-edge (8.960 keV) and post-edge (9.060 keV), respectively. Thin wedge Cu is clearly distinguishable in [(**c**,**d**) insets] from [(**a**,**b**) insets]. (**e**) Line profiles of dotted lines at (**a**) and (**c**), where the thickness of Cu were 300 nm and 50 nm. (**f**) X-ray refractive indices of Cu 100 nm at our scan window. The scale bars are 5 μm.

ZPC-XANES were conducted across the Cu K-absorption edge (8.950–9.070 keV). By increasing the energy by 120 eV, the zone plate moved toward the phase plate approximately 0.9 mm for best focus. If the direct X-rays focus on the back focal plane of 8.950 keV, the beam size variation on that plane at 9.070 eV is 0.695 μm which is smaller than our 4 μm hole of the phase plate (Fig. 1a, inset). The actual phase shift of the phase plate is $\pi$/2+0.076 rad. at 8.950 keV. Although this is not an ideal condition for ZPC, such a small discrepancy does not significantly affect the phase contrast enhancement. In addition, the phase shift variation of the phase plate in the scan range is 0.024 rad, which is considerably smaller than $\pi$/2. Considering these aspects, we fixed the position of the phase plate at the Cu K-edge energy and scanned across the absorption edge for XANES spectroscopy. Owing to minimal artifacts such as halo and shade-off^29,30^, and simple alignment of off-axis illumination, we can demonstrate ZPC-XANES. Additionally, the absorption-based conventional TXM-XANES was separately conducted under identical conditions, but without the phase plate.

**Figure 3:**
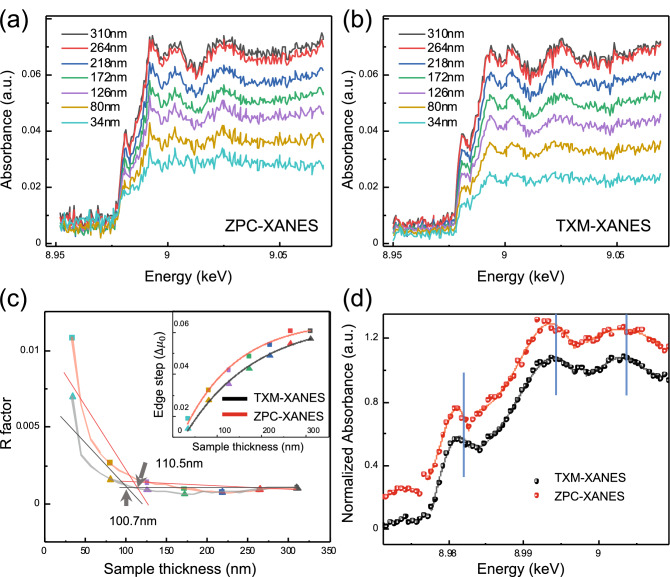
XANES analysis depends on sample thickness (**a**,**b**) XANES spectra of each thickness from ZPC-XANES and TXM-XANES. (**c**) Estimation of minimum thickness for XANES analysis from R factor ($\sum {i}_{\text{(data-fit } )}^{2} / \sum {i}_{\text{(data })}^{2}$). The reasonable minimum thickness is 110.5 nm at ZPC-XANES and 100.7 nm at TXM-XANES (gray arrows). The inset shows the non-linear relationship between the edge step of XANES spectra and Cu thickness. The colors of each dot are related to the colors of thickness from (**a**,**b**). (**d**) Normalized XANES spectra for ZPC (red dots) and TXM-XANES (black dots) at 287 nm thickness. The solid lines indicate the location of the peaks by using a Gaussian curve fitting. The first peaks are at 8981.23 eV (ZPC-XANES) and 8981.56 eV (TXM-XANES). The second peaks are at 8993.68 eV (ZPC-XANES) and 8994.11 eV (TXM-XANES). The third peaks are at 9003.07 eV (ZPC-XANES) and 9003.11 eV (TXM-XANES).

**Figure 4:**
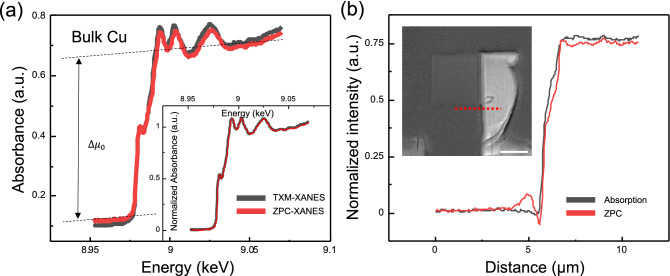
ZPC effects on XANES spectrum and TXM of bulk Cu. (**a**) XANES spectra of bulk Cu with 5 μm thickness. The normalized spectra are demonstrated in the inset. (**b**) Line profile of dotted line at the red line in the inset. The inset shows ZPC TXM at 9.030 keV with a scale bar of 5 μm.

From each energy, one image of the sample and one reference image should be captured to measure absorbance following the Beer–Lambert equation: $\frac{I}{I_{0}}=e^{-\mu t}$, where *I* is the intensity of the transmitted X-rays (image with the sample), $I_{0}$ is the intensity of incident X-rays (image without the sample), $\mu$ is the linear attenuation coefficient of the material, and *t* is the thickness of the material through which X-rays have traveled. When compared to X-ray absorption spectroscopy (XAS), the flat field images are not acquired simultaneously with the sample transmission images, which can affect the final XANES spectra. To reduce the influence of beam motion or instabilities, we selected a set of the projection image and the reference image among five sets of them at each energy that has the minimum standard deviation among sample-free regions in a reference corrected image. By selecting, the noise value was $\sim$ 34.5% better than the worst case (Supplementary Fig. S2). In a conventional absorption-based TXM, it is difficult to observe weak absorption materials (Fig. 2a). At the bottom of the wedge Cu with a thickness of 34 nm, the expected transmission is 0.999 at the pre-edge (8.960 keV) according to the Beer–Lambert equation. Figure 2c shows an evident contrast improvement with ZPC under the same conditions. In contrast to the absorption-based TXM (Fig. 2a, inset), in ZPC TXM (Fig. 2c, inset), the grain boundaries of Cu are distinct, as seen in the electron microscope (Fig. 1b, inset). To confirm the effect of the Zernike phase plate on the image, we investigated the line profiles of wedge Cu with the thickness of 300 nm and 50 nm, as shown in Fig. 2e. The signal-to-noise ratio (SNR) was clearly enhanced with the Zernike phase plate. By the way, at the post-edge, the absorption-based image (Fig. 2b) also makes contrast, but it can be further improved by ZPC (Fig. 2d). The Cu contrast index, which is proportional to the SNR for both absorption and ZPC, was calculated by applying a wave propagation model^18,31^:

1 $$\begin{aligned}&\Theta _{A B S} \approx \frac{2 \pi \sqrt{2} t_{c u}}{\lambda }\left| \beta _{C u}-\beta _{\text{ Air } }\right| e^{-\mu _{\text{ Air } } t_{\text {Air}} / 2} \end{aligned}$$

2 $$\begin{aligned}&\Theta _{Z P C} \approx \frac{2 \pi \sqrt{2} t_{C u}}{\lambda }\left| \delta _{C u}-\delta _{\text{ Air } }\right| e^{-\mu _{\text{ Air } } t_{\text{ Air } } / 2} \end{aligned}$$

where the refractive index of a material traversed by an X-ray beam can be expressed as $n=1-\delta -i \beta$, and the linear attenuation coefficient ($\mu$) is $\frac{4 \pi \beta }{\lambda }$. Figure 2f shows the $\delta$ and $\beta$ for Cu 100 nm at the scan range. This model simulates the X-ray pathway and its interaction with Cu 100 nm and background (air, assumed $\hbox {N}_{{2}}$ with 10 cm). The ZPC provides a contrast index that is 6 times enhanced, which explains the improvement of the SNR in Fig. 2 (e). Although we optimized the Au phase plate by minimizing the hole and adopting off-axis illumination, the phase contrast artifacts, including halo and shade-off, may reduce the difference in intensity^29,30^.

To validate the enhancement of the sensitivity, we investigated the XANES spectra of the wedge Cu region, as shown in Fig. 3. From the series of TXM images, we obtained the XANES spectra with TXM Wizard software^16^. The energy of each XANES scan was calibrated based on the Cu K-edge reference spectrum, which has an edge at 8.979 keV. The XANES spectra are shown unnormalized to highlight the differences in the edge step. The thickness of each wedge point was measured from the SEM image (Fig. 1b, side view). In the XANES spectrum, the size of the edge step ($\Delta \mu _{0}$) is related to the absorber element^32^. In this context, our sample consisted of the same material, and the thickness variation of the sample induced a difference at the edge step, as shown in Fig. 3a for ZPC and Fig. 3b for absorption.

By fitting the spectra from Fig. 3a,b to the Cu reference spectrum, we can quantify the R factor, a commonly used metric for XANES fitting models, for each spectrum to estimate the minimum thickness for XANES analysis in Fig. 3c. The R factor was in the order of $10^{-3}$ (it means ‘good’ fit) in $\ge$ 100 nm thickness (i.e. absorbance $\ge$ 2.2%). These results are the first to experimentally demonstrate the limits of XANES imaging. However, contrary to expectations, the minimum thickness based on the R factor was slightly lower in TXM-XANES than in ZPC-XANES. Comparing both spectra, the standard deviation was large; therefore, the R factor was large in ZPC-XANES. In addition, the peak to valley values of each peak were relatively large. Therefore, each point of the spectrum responded sensitively to the sample or beam fluctuations, which indicates that the sensitivity was improved. In contrast, by comparing the edge step of the spectra (Fig. 3c, inset), the edge steps of ZPC-XANES were approximately 14% higher than those of TXM-XANES at all thicknesses. It is well known that there is a non-linear relationship between thickness and step edge^32^ because the incident beam attenuates as it enters the sample and fluorescence emitted deep inside the sample is more likely to be reabsorbed by the sample. Generally, the goal of XANES spectroscopy is to determine the oxidation state and coordination environment of the element. For instance, the rising edge and the edge maxima shift to higher energies as the oxidation state increases in the case of a similar ligand system. In addition, the coordination number and geometry can be estimated from the rising edge, which has a strong contribution to the 1s–4p transition. Therefore, a 14% enhancement in sensitivity is a significant advance in XANES imaging, particularly in weak absorption material analysis.

However, an undesirable side effect, that is, a slight peak shift near the absorption edge, occurred. Figure 3d shows the representative normalized spectra of ZPC and absorption at 287 nm thickness. (Additionally, spectra at 34 nm thickness are shown in Supplementary Fig. S3). The first and second peaks of ZPC spectra shifted to lower energy by 0.33 eV and 0.43 eV compared to the absorption peaks, respectively. These values were higher than the fitting error. The shift in the third peak was negligible. In addition, the slope of the edge and the width of the peaks of the ZPC-XANES were rather small. A theoretical calculation is required to exactly explain the shift, but we can intuitively infer the reason as the energy dependence of the refractive index that affects the phase contrast. As shown in Fig. 2f, there is an inflection point in the refractive index at the absorption edge energy (8.979 keV). From 8.979 to 9.005 keV, the refractive index increases linearly. That is, the phase of the sample changes linearly. As mentioned earlier, in ZPC, the phase directly corresponds to the intensity of an image. If the sample has a pure phase without absorption, the intensity of an image is higher (in other words, absorption is lower) at 8.979 keV than at 9.005 keV. Therefore, when the sample has both phase and absorption, the absorption, which is linearly changed by ZPC, and the intrinsic absorption (stepwise) of the material should be considered simultaneously. We expect that the absorbance due to these two effects is not simply a linear summation. Because the phase contrast only depends on the refractive index ($\propto$ electron density) of the sample, a peak shift in ZPC-XANES indicates that a combination has occurred. An in-depth theoretical calculation is required, but it is beyond the scope of this study. This will be addressed in a future study.

The basic assumption of the ZPC method is that the phase of the sample is considerably lower than one. In our case, the phase delay of copper was 0.02 rad. (34 nm thick) to 0.18 rad. (310 nm thick) at 8979 eV. Therefore, it can be said that the wedge sample was included within the range of the assumption. Then, a case outside this assumption, that is, very thick samples, was considering. We plotted the XANES spectra of the bulk Cu region (5 μm thick), as shown in Fig. 4a. By selecting the ROI in the bulk Cu region in Fig. 1b, both phase contrast (red) and absorption (black) XANES spectra were obtained. The edge step of each spectrum was taken as the difference at 8.979 keV between a line regressed through the pre-edge (8.941–8.949 keV) region and a quadratic polynomial regressed through the post-edge (8.994–9.061 keV) region of the Cu K-edge XANES spectra. Both spectra are indistinguishable (there is a negligible peak shift within the fitting error) once it is normalized (Fig. 4a, inset). To confirm the effect of the Zernike phase plate on the image, we investigated the line profile of the bulk region, as shown in Fig. 4b. Interestingly, the SNR of the ZPC also decreased. As expected, this means that the effect of ZPC is negligible, and absorption has a major role. However, in contrast to the wedge Cu results, the edge step of ZPC-XANES reduced by approximately $\sim$ 5% compared to TXM-XANES. As described in the wedge sample, the nonlinear relationship between the thickness and step edge in TXM-XANES^32^ is expected to be valid even for thick samples. Therefore, in ZPC-XANES, the step edge is expected to gradually increase as the thickness increases until the ZPC condition is valid and decrease above a certain thickness. To explain the step edge in ZPC-XANES more accurately, a thicker wedge sample and a modified ZPC formula are needed. This will be addressed in a future study.

## Conclusion

We experimentally reported, for the first time, how ZPC affects the XANES spectrum. Using off-axis illumination and a pinhole-type phase plate, we obtained the XANES spectrum using the ZPC method. The phase plate, which did not have an exact $\pi /2$ phase shift and was not exactly on the back focal plane, still enhanced the contrast to weak absorption regions. As a result, the edge steps of the ZPC-XANES spectra were higher than those of the absorption-based TXM-XANES. Furthermore, we were able to estimate the minimum sample absorption for reliable XANES analysis with a specially fabricated wedge-shaped sample. However, this contrast enhancement also affected noise fluctuation and the position of peak locations near the absorption edge where the refractive index significantly changed. Because enhancing the SNR is one of the important challenges in spectroscopic TXM, this study used the ZPC-XANES to guide a possible approach to enhance the contrast of TXM-XANES. Furthermore, it can lighten the non-ideal Zernike phase-contrast method. We used a rather large sized pinhole in the phase plate for ZPC to ensure that it covers all the scan energy. We are sure that the smaller the pinhole size, the better SNR and sensitivity. Further studies are required to validate the performance of our ZPC-XANES in real examples, such as electrode materials with low fractions, and theoretically prove the origin of the energy shift and the non-ideal Zernike phase contrast theoretically.

## Methods

### ZPC-XANES installation

The TXM-XANES setup at BL7C is briefly described below, and more details can be found elsewhere^26–28^. X-rays emitted from the undulator source were monochromatized by a double-crystal Si (111) monochromator in the range of 5–15 keV. The energy of the incoming X-rays was adjusted from 8.950 to 9.070 keV with a 0.5 eV step size. The horizontal and vertical bendable plane mirrors (HFM and VFM) focused the monochromatic beam on the sample position. The incident photon flux at the sample position is $\sim 10^{13} \,{\mathrm {photons}}/200 \upmu {\mathrm {m}}\,({\mathrm {H}}) \times 50\, \upmu {\mathrm {m}}({\mathrm {V}})/{\mathrm {s}}=1 \times 10^{9} {\mathrm {photons}}/\upmu {\mathrm {m}}^{2}/{\mathrm {s}}$ (if we use a capillary condenser, the incident photon flux at the sample position is $\sim 10^{13}\,{\mathrm {photons}}/30\, \upmu {\mathrm {m}}\,({\mathrm {H}}) \times 30\, \upmu {\mathrm {m}}\,({\mathrm {V}})/{\mathrm {s}}=1 \times 10^{10}\,{\mathrm {photons}}/\upmu {\mathrm {m}}^{2}/{\mathrm {s}}.$ Therefore, the incident photon flux can be increased by a factor of 10). The focused beam was monitored using an X-ray beam position monitor (XBPM) with a feedback system linked to the VFM to maintain the beam position along with energy change^33^. A pinhole with a diameter of 50 μm was installed in front of the sample position to define the illumination and avoid zone plate damage. In addition, a diffuser (rotating paper) was inserted next to the pinhole to reduce the spatial coherency and homogenize the illumination. The sample position was controlled using linear stages with an encoding function that needs to obtain flat-field images. After the sample stage with distance “a”, following the Lens formula ($\frac{1}{a}+\frac{1}{b}=\frac{1}{f}$, where *f* is the focal length of the lens), the objective Fresnel zone plate of diameter 300 μm, outermost zone width 30 nm (Applied Nanotools, Canada) produced the $\times \frac{b}{a}$ magnified images of the sample on a scintillator crystal (20 μm thick GaGG) where X-rays were converted to visible light. A phase plate with a 4 μm diameter hole on a gold film phase plate with a 958.8 nm thickness (Luxel, USA) was inserted at the back focal plane of the Fresnel zone plate. The optimum energy that satisfies the Zernike phase-contrast condition ($\pi$/2 phase shift by the phase plate), is 9.332 keV. The resulting visible image was magnified with $\times 20$ optical objective lens and captured by a 16-bit $2,048 \times 2,048$ pixels CCD camera (Princeton Instrument, USA), which was placed at “b” distance from Fresnel zone plate. The FOV was 43 μm for all experiments.

### Sample preparation

The Cu microparticles were purchased from Sigma-Aldrich. A Cu microparticle within the FOV was chosen to cover the entire sample at one projection. Furthermore, to measure the resolution of the detection thickness, we conducted focused ion beam (FIB) milling to obtain wedge structures. As a result, we obtained our sample with two sections, as shown in Fig. 1b: Wedge Cu with gradually declined thickness from 310 to 34 nm, and bulk Cu with a thickness of 5 μm along the beam direction.

### Image acquisition and data processing

A long region [10 μm (H) $\times$ 0.4 μm (V)] was selected to see the difference in spectra by thickness (Supplementary Fig. S4). To investigate the effect of ZPC’s artifacts on the XANES spectrum, two regions were selected. The region with halo artifacts shows relatively low edge step and low sensitivity compared to the artifacts-free region. The halo artifacts lower the sensitivity and edge step of the XANES spectrum. To reduce the influence of beam motion or instabilities, we selected a set of projection images and reference images among five sets of images at each energy. One reference corrected image with the lowest standard deviation of pixel intensity from the empty region (no object) was selected from the five reference corrected images at each energy level (Supplementary Fig. S2). The selection was performed using the Python code. After the selection, the selected data went through several image processing steps by TXM-wizard^16^ including the reference correction, magnification correction, and spatial registration of images taken at different energies. The corrected images were divided into bulk Cu and extracted Cu at each thickness with 10 pixels height from the wedge structure. After extracting spectra from divided images, the spectra were subjected to spectroscopic analysis using a software package known as Athena^34^ for a more detailed investigation, including calibration, normalization, and measuring edge step. Finally, a least-square fitting method was used to evaluate the extracted Cu spectra, which were fitted to the bulk Cu spectrum using the SIX Pack software^35^.

## Supplementary Information

Supplementary Figures.
