# Supplementary material for: Exploring spectroscopic X-ray nano-imaging with Zernike phase contrast enhancement
Source: Sci Rep. 2022 Feb 21;12:2894. doi: 10.1038/s41598-022-06827-y (PMC8861036; doi:10.1038/s41598-022-06827-y)
Supplement: Supplementary file 1 — Supplementary Figures. [file 41598_2022_6827_MOESM1_ESM.pdf]

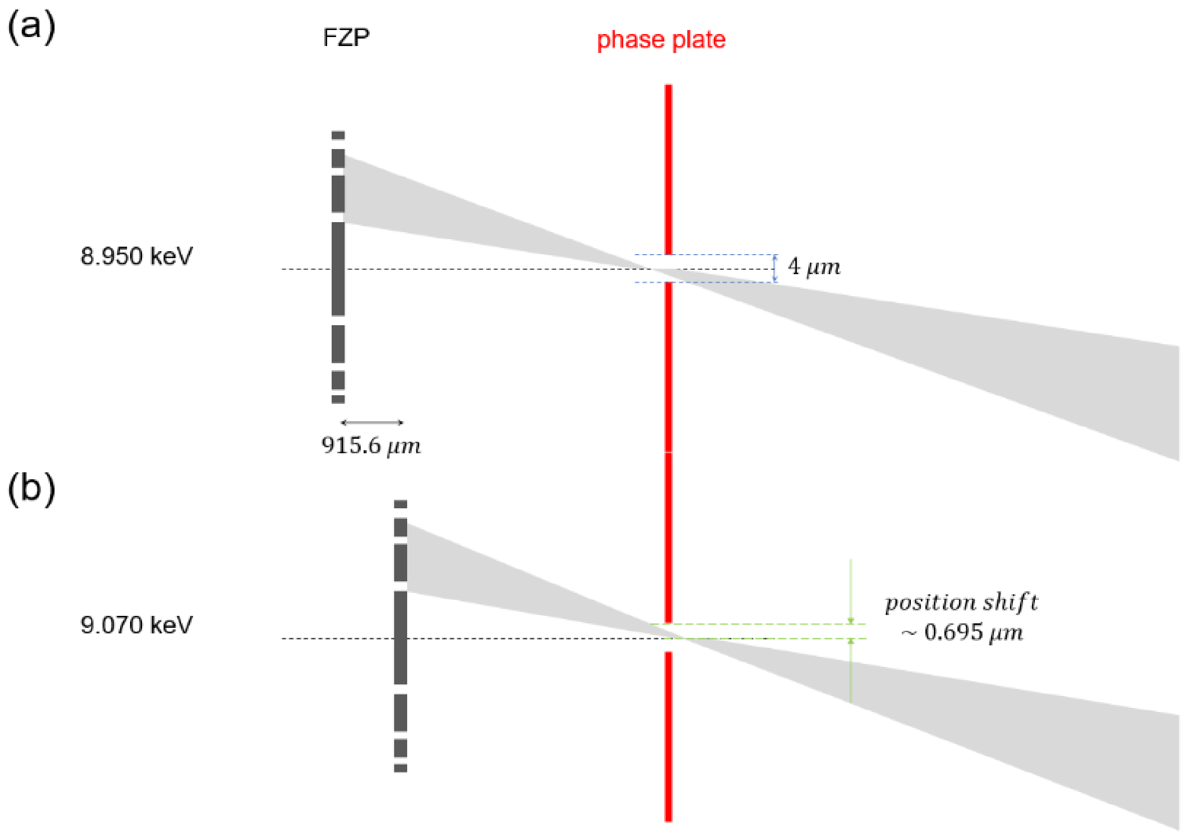

Supplementary Figure S1. Position shift of the focused beam on the plane of the phase plate (a) at 8.950 keV (b) at 9.070 keV.

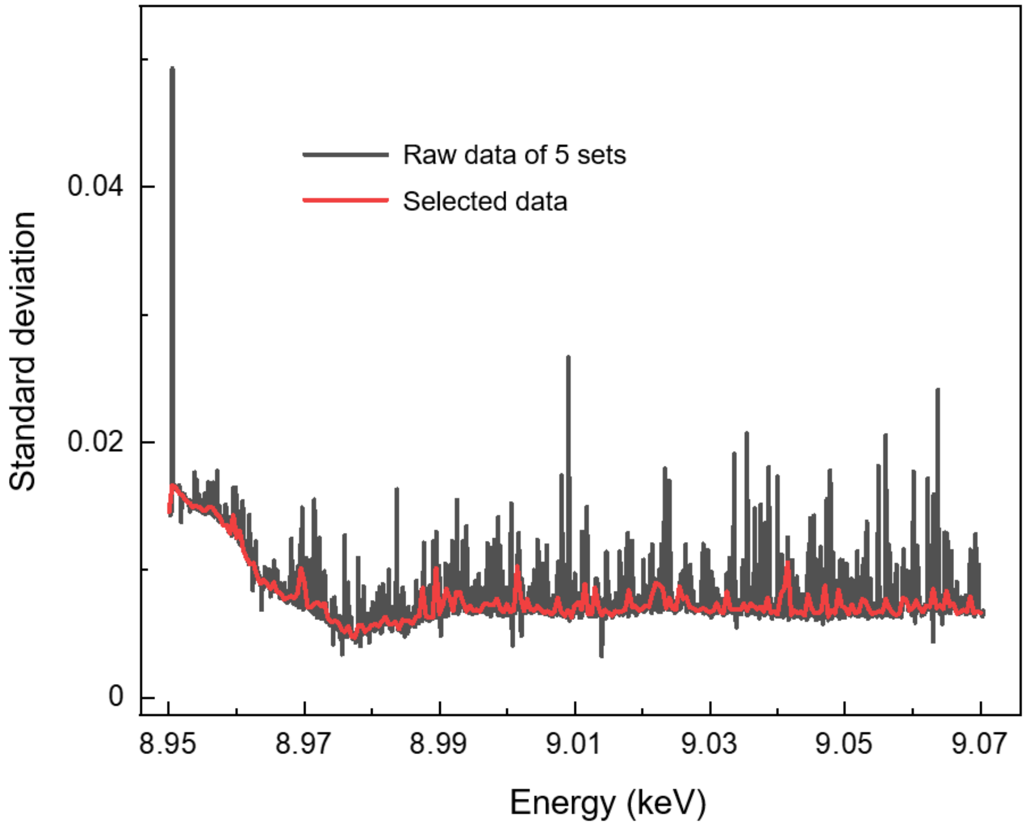

Supplementary Figure S2. Intensity standard deviations of empty region without object (background corrected) across all scan range.

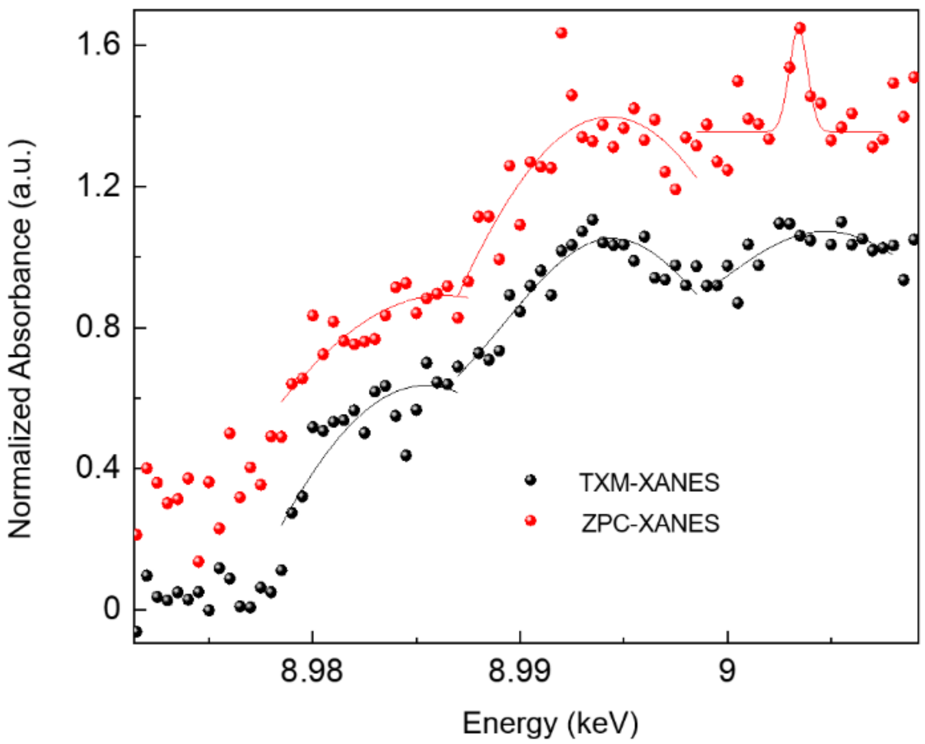

Supplementary Figure S3. Normalized XANES spectra for ZPC (red dots) and TXM-XANES (black dots) at 34 nm thickness. The solid lines indicate the location of the peaks by using a Gaussian curve fitting. Both methods show low SNR values and rather noisy spectra due to low absorbance. It is hard to define the peak positions.

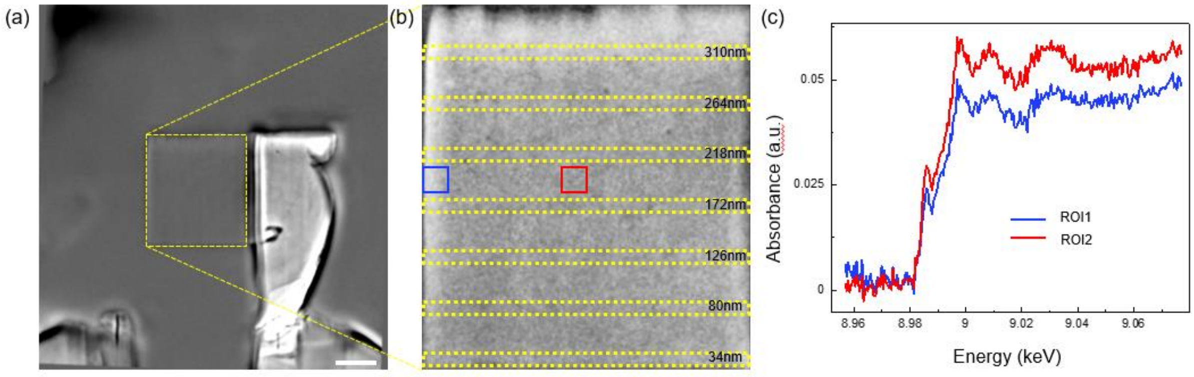

Supplementary Figure S4. Halo artifacts effect on XANES spectrum. (a) Sample image, (b) sampling area of each thickness, and (c) XANES spectrum of halo artifacts region (ROI 1) and artifacts free region (ROI 2).
